# Bis(4,4′′-difluoro-1,1′:3′,1′′-terphenyl-2′-carboxyl­ato-κ*O*)tetra­kis­(methanol-κ*O*)calcium methanol tetra­solvate

**DOI:** 10.1107/S1600536813000044

**Published:** 2013-01-23

**Authors:** Namseok Kim, Heeso Noh, Sungho Yoon, Chan Ryang Park

**Affiliations:** a Department of Bio & Nano Chemistry, College of Natural Sciences, Kookmin University, 861-1 Jeongneung-dong, Seongbuk-gu, Seoul 136-702, Republic of Korea; b Department of Physics, College of Natural Sciences, Kookmin University, 861-1 Jeongneung-dong, Seongbuk-gu, Seoul 136-702, Republic of Korea.

## Abstract

In the title compound, [Ca(C_19_H_11_F_2_O_2_)_2_(CH_3_OH)_4_]·4CH_3_OH, the Ca^2+^ ion is located on an inversion centre and is hexa­coordinated by two O atoms of two 4,4′′-difluoro-1,1′:3′,1′′-terphenyl-2′-carboxyl­ate ligands and four O atoms of four methanol ligands, forming a CaO_6_ polyhedron with a slightly distorted octa­hedral coordination geometry. The Ca—O—C angle between the carboxyl­ate group and the calcium ion is 171.8 (2)°. Two types of inter­molecular hydrogen-bond inter­actions (C=O⋯H and O—H⋯O) between the carboxyl­ate ligand, the methanol solvent mol­ecules and the coordinating methanol ligands generate a two-dimensional network parallel to (001).

## Related literature

For background to metal complexes with 4,4′′-difluoro-1,1′:3′,1′′-terphenyl-2′-carboxyl­ate ligands, see: Kannan *et al.* (2011 ▶); Chavez *et al.* (2001 ▶). For mononuclear calcium complexes with carboxyl­ate ligands, see: Perrin *et al.* (2009 ▶); Godino Salido *et al.* (2004 ▶); Huang *et al.* (2010 ▶). For their polymerization behavior, see: Jisha *et al.* (2010 ▶); Murugavel & Banerjee (2003 ▶); Yang *et al.* (2004 ▶). 
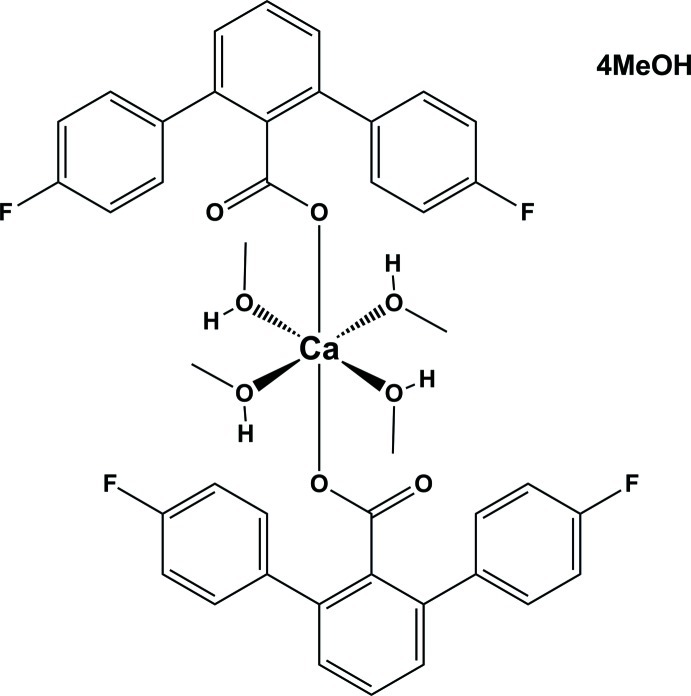


## Experimental

### 

#### Crystal data

[Ca(C_19_H_11_F_2_O_2_)_2_(CH_4_O)_4_]·4CH_4_O
*M* *_r_* = 914.97
Orthorhombic, 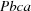

*a* = 15.4611 (19) Å
*b* = 14.2436 (18) Å
*c* = 20.886 (3) Å
*V* = 4599.5 (10) Å^3^
*Z* = 4
Mo *K*α radiation
μ = 0.21 mm^−1^
*T* = 200 K
0.32 × 0.23 × 0.14 mm

#### Data collection

Bruker SMART CCD area-detector diffractometer
Absorption correction: multi-scan (*SADABS*; Bruker, 2000 ▶) *T*_min_ = 0.521, *T*_max_ = 1.00
32627 measured reflections
5709 independent reflections
2624 reflections with *I* > 2σ(*I*)
*R*_int_ = 0.143

#### Refinement

*R*[*F* ^2^ > 2σ(*F* ^2^)] = 0.064
*wR*(*F* ^2^) = 0.165
*S* = 0.98
5709 reflections
300 parameters
H atoms treated by a mixture of independent and constrained refinement
Δρ_max_ = 0.43 e Å^−3^
Δρ_min_ = −0.45 e Å^−3^

### 

Data collection: *SMART* (Bruker, 2000 ▶); cell refinement: *SAINT* (Bruker, 2000 ▶); data reduction: *SAINT*; program(s) used to solve structure: *SHELXS97* (Sheldrick, 2008 ▶); program(s) used to refine structure: *SHELXL97* (Sheldrick, 2008 ▶); molecular graphics: *SHELXTL* (Sheldrick, 2008 ▶); software used to prepare material for publication: *SHELXTL*.

## Supplementary Material

Crystal structure: contains datablock(s) I, global. DOI: 10.1107/S1600536813000044/ru2047sup1.cif

Structure factors: contains datablock(s) I. DOI: 10.1107/S1600536813000044/ru2047Isup2.hkl

Additional supplementary materials:  crystallographic information; 3D view; checkCIF report

## Figures and Tables

**Table 1:** Selected bond lengths (Å)

Ca1—O1	2.2693 (19)
Ca1—O4	2.325 (2)
Ca1—O3	2.346 (2)

**Table 2:** Hydrogen-bond geometry (Å, °)

*D*—H⋯*A*	*D*—H	H⋯*A*	*D*⋯*A*	*D*—H⋯*A*
O3—H1⋯O6^ii^	0.89 (4)	1.78 (4)	2.636 (3)	162 (4)
O4—H2⋯O5^iii^	0.91 (4)	1.76 (4)	2.660 (3)	172 (4)
O6—H6*A*⋯O2^iv^	0.84	1.96	2.804 (3)	177
O5—H5*A*⋯O2^v^	0.84	1.92	2.755 (3)	170

Symmetry codes: (ii) 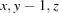; (iii) 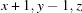; (iv) 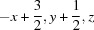; (v) 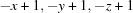.

**Fig. 1.**
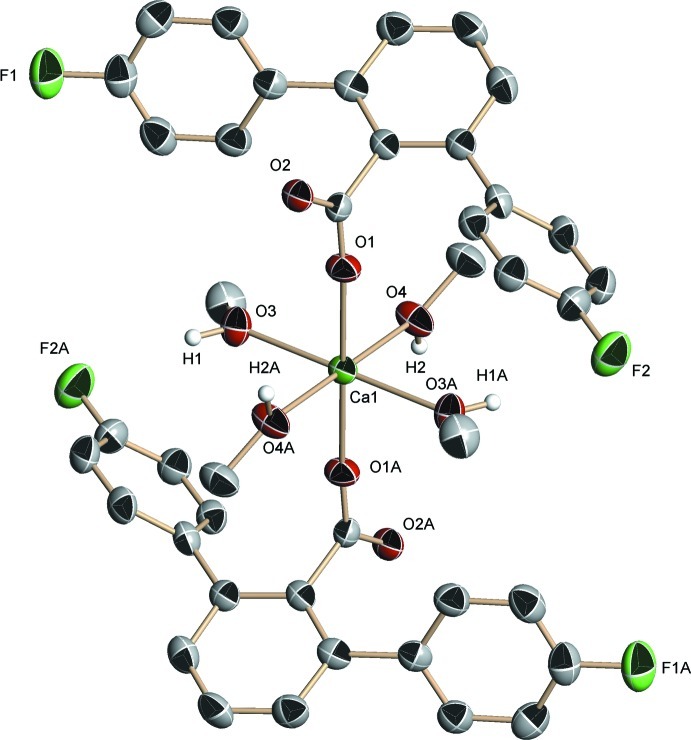
The molecular structure of the title compound, showing the atom-numbering and with displacement ellipsoids drawn at the 50% probability level.

**Fig. 2.**
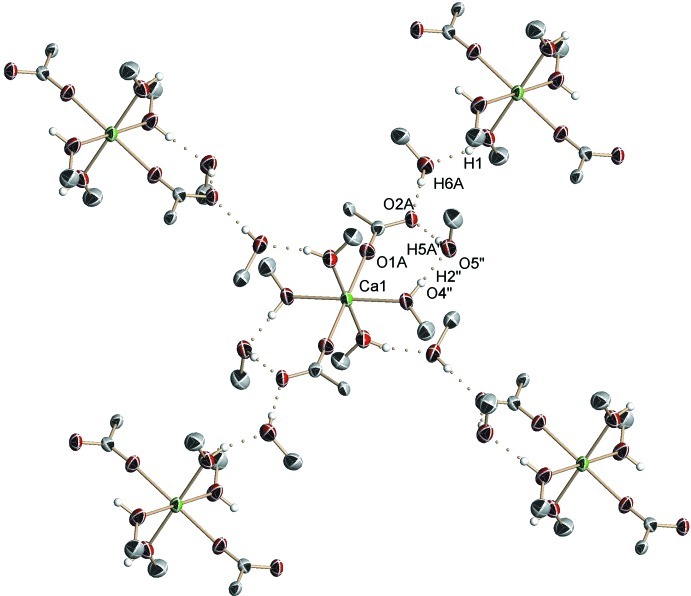
A crystal packing diagram of the title compound, showing the hydrogen bonds and with displacement ellipsoids drawn at the 50% probability level.

## supplementary crystallographic information

### Comment

Octahedral mononuclear transition metal complexes with ligated carboxylates are well known structure for basic of inorganic chemistry (Chavez *et al.*, 2001; Kannan *et al.*, 2011). Few mononuclear calcium complexes with carboxylate ligands (Perrin *et al.*, 2009; Godino Salido *et al.*, 2004; Huang *et al.*, 2010) are reported possible due to easy polymerization behavior (Murugavel *et al.*, 2003; Jisha *et al.*, 2010; Yang *et al.*, 2004). Here, we report the structure of an octahedrally coordinated Ca^2+^ complex which crystallizes in the orthorhombic space group *Pbca* with one half molecule in the asymmetric unit. The selected bond distances and angles of [Ca(C_19_H_11_O_2_F_2_)_2_(CH_4_O)_4_] are given in Table 1 with the structure of the molecule shown, in Fig 1, and its crystal packing involving strong intermolecular C=O···H, O—H···O interactions are detailed in Fig 2 and Table 2.

### Experimental

To a solution of sodium 4,4''-difluoro-1,1':3',1''-terphenyl-2'-carboxylate (0.200 g, 0.602 mmol) in 15 ml of methanol, Ca(CF_3_SO_3_)_2_ (0.204 g, 0.602 mmol) was added at room temperature. After stirring for 30 min, colorless block type crystals were collected from slow evaporization. Yield = 51%, (0.281 g).

### Refinement

H atoms were placed at calculated positions and refined as riding with C–H(aromatic) = 0.95 Å, C–H(CH_3_) = 0.98 Å, and with *U*_iso_(H) = 1.2 *U*_eq_(C) or 1.5 *U*_eq_(C) for methyl groups. The O-bound H atoms of methanol were located in a difference Fourier map and refined isotropically.

### Crystal data

**Table:**

[Ca(C_19_H_11_F_2_O_2_)_2_(CH_4_O)_4_]·4CH_4_O	*F*(000) = 1928
*M**_r_* = 914.97	*D*_x_ = 1.321 Mg m^−^^3^
Orthorhombic, *P**b**c**a*	Mo *K*α radiation, λ = 0.71073 Å
Hall symbol: -p 2ac 2ab	Cell parameters from 2898 reflections
*a* = 15.4611 (19) Å	θ = 2.2–22.0°
*b* = 14.2436 (18) Å	µ = 0.21 mm^−^^1^
*c* = 20.886 (3) Å	*T* = 200 K
*V* = 4599.5 (10) Å^3^	Block, colorless
*Z* = 4	0.32 × 0.23 × 0.14 mm

### Data collection

**Table:**

Bruker SMART CCD area-detector diffractometer	5709 independent reflections
Radiation source: fine-focus sealed tube	2624 reflections with *I* > 2σ(*I*)
Graphite monochromator	*R*_int_ = 0.143
phi and ω scans	θ_max_ = 28.3°, θ_min_ = 2.0°
Absorption correction: multi-scan (*SADABS*; Bruker, 2000)	*h* = −14→20
*T*_min_ = 0.521, *T*_max_ = 1.00	*k* = −18→18
32627 measured reflections	*l* = −23→27

### Refinement

**Table:**

Refinement on *F*^2^	Primary atom site location: structure-invariant direct methods
Least-squares matrix: full	Secondary atom site location: difference Fourier map
*R*[*F*^2^ > 2σ(*F*^2^)] = 0.064	Hydrogen site location: inferred from neighbouring sites
*wR*(*F*^2^) = 0.165	H atoms treated by a mixture of independent and constrained refinement
*S* = 0.98	*w* = 1/[σ^2^(*F*_o_^2^) + (0.0644*P*)^2^] where *P* = (*F*_o_^2^ + 2*F*_c_^2^)/3
5709 reflections	(Δ/σ)_max_ < 0.001
300 parameters	Δρ_max_ = 0.43 e Å^−^^3^
0 restraints	Δρ_min_ = −0.45 e Å^−^^3^

### Special details

**Table:**

Geometry. All e.s.d.'s (except the e.s.d. in the dihedral angle between two l.s. planes) are estimated using the full covariance matrix. The cell e.s.d.'s are taken into account individually in the estimation of e.s.d.'s in distances, angles and torsion angles; correlations between e.s.d.'s in cell parameters are only used when they are defined by crystal symmetry. An approximate (isotropic) treatment of cell e.s.d.'s is used for estimating e.s.d.'s involving l.s. planes.
Refinement. Refinement of *F*^2^ against ALL reflections. The weighted *R*-factor *wR* and goodness of fit *S* are based on *F*^2^, conventional *R*-factors *R* are based on *F*, with *F* set to zero for negative *F*^2^. The threshold expression of *F*^2^ > σ(*F*^2^) is used only for calculating *R*-factors(gt) *etc*. and is not relevant to the choice of reflections for refinement. *R*-factors based on *F*^2^ are statistically about twice as large as those based on *F*, and *R*- factors based on ALL data will be even larger.

### Fractional atomic coordinates and isotropic or equivalent isotropic displacement parameters (Å^2^)

**Table:**

	*x*	*y*	*z*	*U*_iso_*/*U*_eq_	
Ca1	1.0000	0.0000	0.5000	0.0296 (2)	
O2	0.79691 (12)	0.20766 (14)	0.46541 (10)	0.0359 (5)	
O1	0.92097 (13)	0.12979 (14)	0.47660 (10)	0.0382 (6)	
O3	0.94225 (15)	−0.07389 (16)	0.40917 (11)	0.0416 (6)	
O4	1.12075 (14)	0.04631 (15)	0.44165 (11)	0.0436 (6)	
F2	1.02858 (13)	0.35764 (16)	0.71251 (10)	0.0657 (6)	
F1	0.68180 (14)	0.03347 (16)	0.22211 (11)	0.0698 (7)	
C15	0.7834 (2)	0.2459 (2)	0.27894 (15)	0.0414 (8)	
H15	0.7766	0.3120	0.2754	0.050*	
C1	0.87736 (19)	0.1973 (2)	0.45564 (14)	0.0293 (7)	
C8	0.99166 (19)	0.3377 (2)	0.51732 (15)	0.0349 (8)	
C2	0.92369 (18)	0.27084 (19)	0.41636 (15)	0.0297 (7)	
C6	0.9544 (2)	0.3437 (2)	0.31427 (17)	0.0439 (9)	
H6	0.9460	0.3476	0.2693	0.053*	
C14	0.8506 (2)	0.2098 (2)	0.31613 (15)	0.0353 (8)	
C3	0.97877 (18)	0.3349 (2)	0.44720 (16)	0.0327 (8)	
C7	0.91018 (19)	0.2756 (2)	0.35003 (15)	0.0344 (8)	
C5	1.0100 (2)	0.4050 (2)	0.34434 (17)	0.0455 (9)	
H5	1.0407	0.4501	0.3197	0.055*	
C9	0.9219 (2)	0.3391 (2)	0.56012 (16)	0.0368 (8)	
H9	0.8647	0.3351	0.5436	0.044*	
C13	1.0747 (2)	0.3425 (2)	0.54382 (17)	0.0400 (8)	
H13	1.1235	0.3414	0.5161	0.048*	
C19	0.8596 (2)	0.1135 (2)	0.31971 (16)	0.0451 (9)	
H19	0.9049	0.0876	0.3448	0.054*	
C10	0.9335 (2)	0.3462 (2)	0.62520 (16)	0.0428 (8)	
H10	0.8855	0.3479	0.6535	0.051*	
C4	1.0216 (2)	0.4017 (2)	0.40898 (18)	0.0426 (9)	
H4	1.0595	0.4455	0.4287	0.051*	
C11	1.0168 (2)	0.3508 (2)	0.64801 (17)	0.0454 (9)	
C12	1.0876 (2)	0.3486 (2)	0.60847 (17)	0.0439 (9)	
H12	1.1444	0.3512	0.6256	0.053*	
C17	0.7379 (2)	0.0923 (3)	0.25238 (17)	0.0479 (9)	
C21	1.1492 (2)	0.1374 (2)	0.42276 (18)	0.0514 (10)	
H21A	1.1019	0.1703	0.4011	0.077*	
H21B	1.1984	0.1314	0.3934	0.077*	
H21C	1.1669	0.1730	0.4607	0.077*	
C20	0.9850 (2)	−0.1026 (3)	0.35239 (19)	0.0562 (10)	
H20A	1.0103	−0.0477	0.3312	0.084*	
H20B	0.9433	−0.1327	0.3236	0.084*	
H20C	1.0309	−0.1474	0.3632	0.084*	
C18	0.8036 (2)	0.0540 (2)	0.28735 (17)	0.0503 (10)	
H18	0.8108	−0.0122	0.2895	0.060*	
C16	0.7266 (2)	0.1872 (3)	0.24715 (16)	0.0472 (9)	
H16	0.6808	0.2123	0.2222	0.057*	
O5	0.24558 (14)	0.91787 (15)	0.43929 (12)	0.0451 (6)	
H5A	0.2373	0.8828	0.4711	0.068*	
O6	0.78040 (14)	0.86827 (15)	0.41295 (12)	0.0483 (6)	
H6A	0.7576	0.8192	0.4275	0.073*	
C23	0.7269 (2)	0.9463 (2)	0.42684 (19)	0.0504 (10)	
H23A	0.7059	0.9417	0.4710	0.076*	
H23B	0.6775	0.9468	0.3974	0.076*	
H23C	0.7601	1.0044	0.4217	0.076*	
C22	0.2469 (2)	0.8631 (3)	0.38278 (18)	0.0554 (10)	
H22A	0.2503	0.9045	0.3454	0.083*	
H22B	0.1940	0.8254	0.3803	0.083*	
H22C	0.2974	0.8215	0.3835	0.083*	
H2	1.163 (2)	0.003 (3)	0.4370 (19)	0.078 (13)*	
H1	0.892 (3)	−0.104 (3)	0.4134 (19)	0.084 (15)*	

### Atomic displacement parameters (Å^2^)

**Table:**

	*U*^11^	*U*^22^	*U*^33^	*U*^12^	*U*^13^	*U*^23^
Ca1	0.0215 (4)	0.0302 (4)	0.0370 (5)	0.0015 (4)	0.0028 (4)	0.0044 (4)
O2	0.0218 (12)	0.0384 (12)	0.0474 (15)	0.0003 (9)	0.0029 (9)	0.0063 (11)
O1	0.0323 (13)	0.0324 (12)	0.0498 (15)	0.0075 (10)	−0.0001 (10)	0.0092 (11)
O3	0.0317 (14)	0.0506 (15)	0.0424 (15)	−0.0045 (12)	0.0031 (10)	−0.0080 (11)
O4	0.0311 (13)	0.0363 (13)	0.0635 (17)	0.0029 (11)	0.0164 (11)	0.0084 (12)
F2	0.0575 (14)	0.0938 (17)	0.0456 (15)	−0.0085 (12)	−0.0073 (10)	−0.0017 (12)
F1	0.0726 (16)	0.0703 (15)	0.0663 (16)	−0.0189 (12)	−0.0069 (12)	−0.0205 (12)
C15	0.049 (2)	0.0419 (19)	0.034 (2)	−0.0009 (17)	0.0049 (16)	0.0006 (16)
C1	0.0267 (18)	0.0323 (16)	0.0289 (18)	−0.0018 (14)	−0.0024 (13)	−0.0011 (14)
C8	0.0302 (18)	0.0283 (16)	0.046 (2)	−0.0047 (14)	0.0023 (15)	−0.0028 (14)
C2	0.0221 (16)	0.0286 (16)	0.039 (2)	0.0030 (13)	0.0053 (13)	0.0005 (14)
C6	0.046 (2)	0.048 (2)	0.038 (2)	−0.0018 (17)	0.0053 (16)	0.0051 (17)
C14	0.038 (2)	0.0380 (18)	0.0298 (19)	0.0003 (15)	0.0071 (14)	0.0016 (15)
C3	0.0244 (17)	0.0344 (17)	0.039 (2)	0.0024 (14)	0.0027 (14)	0.0016 (15)
C7	0.0326 (19)	0.0334 (17)	0.037 (2)	0.0036 (14)	0.0092 (14)	0.0017 (15)
C5	0.050 (2)	0.0404 (19)	0.046 (2)	−0.0122 (17)	0.0131 (17)	0.0055 (17)
C9	0.0301 (19)	0.0355 (18)	0.045 (2)	0.0006 (14)	0.0005 (15)	−0.0024 (15)
C13	0.0250 (18)	0.0425 (19)	0.052 (2)	−0.0040 (15)	0.0025 (15)	−0.0006 (17)
C19	0.055 (2)	0.0374 (19)	0.043 (2)	0.0013 (17)	−0.0025 (17)	0.0010 (16)
C10	0.036 (2)	0.047 (2)	0.045 (2)	0.0004 (15)	0.0014 (16)	−0.0009 (17)
C4	0.035 (2)	0.0391 (19)	0.053 (2)	−0.0065 (15)	0.0053 (16)	−0.0005 (17)
C11	0.050 (2)	0.049 (2)	0.038 (2)	−0.0034 (17)	−0.0063 (17)	−0.0002 (17)
C12	0.032 (2)	0.051 (2)	0.049 (2)	−0.0045 (16)	−0.0059 (16)	−0.0017 (18)
C17	0.049 (2)	0.056 (2)	0.039 (2)	−0.0108 (19)	0.0065 (17)	−0.015 (2)
C21	0.051 (2)	0.044 (2)	0.060 (3)	−0.0037 (18)	0.0096 (18)	0.0131 (18)
C20	0.053 (2)	0.053 (2)	0.062 (3)	0.0039 (19)	0.0097 (19)	−0.011 (2)
C18	0.065 (3)	0.037 (2)	0.049 (2)	−0.0060 (19)	0.0053 (19)	−0.0065 (18)
C16	0.047 (2)	0.059 (2)	0.036 (2)	0.0054 (18)	−0.0005 (17)	−0.0051 (18)
O5	0.0352 (13)	0.0449 (14)	0.0553 (17)	0.0028 (11)	0.0104 (12)	0.0055 (12)
O6	0.0331 (14)	0.0410 (13)	0.0710 (18)	−0.0088 (11)	0.0044 (11)	0.0040 (13)
C23	0.045 (2)	0.039 (2)	0.067 (3)	−0.0086 (17)	0.0080 (18)	0.0013 (18)
C22	0.054 (2)	0.060 (2)	0.052 (3)	0.006 (2)	0.0038 (18)	0.001 (2)

### Geometric parameters (Å, º)

**Table:**

Ca1—O1^i^	2.2692 (19)	C9—C10	1.375 (4)
Ca1—O1	2.2693 (19)	C9—H9	0.9500
Ca1—O4^i^	2.325 (2)	C13—C12	1.368 (4)
Ca1—O4	2.325 (2)	C13—H13	0.9500
Ca1—O3	2.346 (2)	C19—C18	1.388 (4)
Ca1—O3^i^	2.346 (2)	C19—H19	0.9500
O2—C1	1.269 (3)	C10—C11	1.375 (5)
O1—C1	1.253 (3)	C10—H10	0.9500
O3—C20	1.418 (4)	C4—H4	0.9500
O3—H1	0.89 (4)	C11—C12	1.371 (5)
O4—C21	1.425 (4)	C12—H12	0.9500
O4—H2	0.91 (4)	C17—C18	1.364 (5)
F2—C11	1.363 (4)	C17—C16	1.367 (5)
F1—C17	1.362 (4)	C21—H21A	0.9800
C15—C16	1.382 (5)	C21—H21B	0.9800
C15—C14	1.395 (4)	C21—H21C	0.9800
C15—H15	0.9500	C20—H20A	0.9800
C1—C2	1.511 (4)	C20—H20B	0.9800
C8—C13	1.400 (4)	C20—H20C	0.9800
C8—C9	1.401 (4)	C18—H18	0.9500
C8—C3	1.479 (4)	C16—H16	0.9500
C2—C7	1.403 (4)	O5—C22	1.415 (4)
C2—C3	1.404 (4)	O5—H5A	0.8400
C6—C5	1.377 (5)	O6—C23	1.416 (4)
C6—C7	1.403 (4)	O6—H6A	0.8400
C6—H6	0.9500	C23—H23A	0.9800
C14—C19	1.380 (4)	C23—H23B	0.9800
C14—C7	1.492 (4)	C23—H23C	0.9800
C3—C4	1.408 (4)	C22—H22A	0.9800
C5—C4	1.363 (5)	C22—H22B	0.9800
C5—H5	0.9500	C22—H22C	0.9800
			
O1^i^—Ca1—O1	180.00 (8)	C12—C13—H13	119.1
O1^i^—Ca1—O4^i^	95.07 (8)	C8—C13—H13	119.1
O1—Ca1—O4^i^	84.93 (8)	C14—C19—C18	121.2 (3)
O1^i^—Ca1—O4	84.93 (8)	C14—C19—H19	119.4
O1—Ca1—O4	95.07 (8)	C18—C19—H19	119.4
O4^i^—Ca1—O4	180.0	C9—C10—C11	117.9 (3)
O1^i^—Ca1—O3	90.78 (8)	C9—C10—H10	121.0
O1—Ca1—O3	89.22 (8)	C11—C10—H10	121.0
O4^i^—Ca1—O3	89.48 (8)	C5—C4—C3	121.5 (3)
O4—Ca1—O3	90.52 (8)	C5—C4—H4	119.2
O1^i^—Ca1—O3^i^	89.22 (8)	C3—C4—H4	119.2
O1—Ca1—O3^i^	90.78 (8)	F2—C11—C12	119.4 (3)
O4^i^—Ca1—O3^i^	90.52 (8)	F2—C11—C10	118.1 (3)
O4—Ca1—O3^i^	89.48 (8)	C12—C11—C10	122.5 (3)
O3—Ca1—O3^i^	180.0	C13—C12—C11	118.7 (3)
C1—O1—Ca1	171.8 (2)	C13—C12—H12	120.6
C20—O3—Ca1	128.9 (2)	C11—C12—H12	120.6
C20—O3—H1	110 (3)	F1—C17—C18	118.4 (3)
Ca1—O3—H1	118 (3)	F1—C17—C16	119.3 (3)
C21—O4—Ca1	130.6 (2)	C18—C17—C16	122.2 (3)
C21—O4—H2	112 (2)	O4—C21—H21A	109.5
Ca1—O4—H2	116 (2)	O4—C21—H21B	109.5
C16—C15—C14	121.2 (3)	H21A—C21—H21B	109.5
C16—C15—H15	119.4	O4—C21—H21C	109.5
C14—C15—H15	119.4	H21A—C21—H21C	109.5
O1—C1—O2	124.1 (3)	H21B—C21—H21C	109.5
O1—C1—C2	117.8 (3)	O3—C20—H20A	109.5
O2—C1—C2	118.1 (3)	O3—C20—H20B	109.5
C13—C8—C9	117.0 (3)	H20A—C20—H20B	109.5
C13—C8—C3	121.1 (3)	O3—C20—H20C	109.5
C9—C8—C3	121.9 (3)	H20A—C20—H20C	109.5
C7—C2—C3	120.8 (3)	H20B—C20—H20C	109.5
C7—C2—C1	119.9 (3)	C17—C18—C19	118.7 (3)
C3—C2—C1	119.3 (3)	C17—C18—H18	120.6
C5—C6—C7	120.0 (3)	C19—C18—H18	120.6
C5—C6—H6	120.0	C17—C16—C15	118.6 (3)
C7—C6—H6	120.0	C17—C16—H16	120.7
C19—C14—C15	118.1 (3)	C15—C16—H16	120.7
C19—C14—C7	122.4 (3)	C22—O5—H5A	109.5
C15—C14—C7	119.5 (3)	C23—O6—H6A	109.5
C2—C3—C4	117.7 (3)	O6—C23—H23A	109.5
C2—C3—C8	123.6 (3)	O6—C23—H23B	109.5
C4—C3—C8	118.7 (3)	H23A—C23—H23B	109.5
C6—C7—C2	119.1 (3)	O6—C23—H23C	109.5
C6—C7—C14	118.9 (3)	H23A—C23—H23C	109.5
C2—C7—C14	122.0 (3)	H23B—C23—H23C	109.5
C4—C5—C6	120.8 (3)	O5—C22—H22A	109.5
C4—C5—H5	119.6	O5—C22—H22B	109.5
C6—C5—H5	119.6	H22A—C22—H22B	109.5
C10—C9—C8	122.1 (3)	O5—C22—H22C	109.5
C10—C9—H9	119.0	H22A—C22—H22C	109.5
C8—C9—H9	119.0	H22B—C22—H22C	109.5
C12—C13—C8	121.8 (3)		

Symmetry code: (i) −*x*+2, −*y*, −*z*+1.

### Hydrogen-bond geometry (Å, º)

**Table:**

*D*—H···*A*	*D*—H	H···*A*	*D*···*A*	*D*—H···*A*
O3—H1···O6^ii^	0.89 (4)	1.78 (4)	2.636 (3)	162 (4)
O4—H2···O5^iii^	0.91 (4)	1.76 (4)	2.660 (3)	172 (4)
O6—H6*A*···O2^iv^	0.84	1.96	2.804 (3)	177
O5—H5*A*···O2^v^	0.84	1.92	2.755 (3)	170

Symmetry codes: (ii) *x*, *y*−1, *z*; (iii) *x*+1, *y*−1, *z*; (iv) −*x*+3/2, *y*+1/2, *z*; (v) −*x*+1, −*y*+1, −*z*+1.

## References

[bb1] Bruker (2000). *SMART*, *SAINT* and *SADABS* Bruker AXS Inc., Madison, Wisconsin, USA.

[bb2] Chavez, F. A., Que, L. & Tolman, W. B. (2001). *Chem. Commun.* pp. 111–112.

[bb3] Godino Salido, M. L., Arranz Mascarós, P., López Garzón, R., Gutiérrez Valero, M. D., Low, J. N., Gallagher, J. F. & Glidewell, C. (2004). *Acta Cryst.* B**60**, 46–64. 10.1107/S0108768103029136 14734844

[bb4] Huang, L., Zhong, A. G., Chen, D. B., Qiu, D. & Liang, H. D. (2010). *J. Mol. Struct.* **984**, 39–50.

[bb5] Jisha, K. R., Suma, S. & Sudarsanakumar, M. R. (2010). *Polyhedron*, **29**, 3164–3169.

[bb6] Kannan, S., Venkatachalam, G., Lee, H.-J., Kim, W., Koo, E., Do, Y. R. & Yoon, S. (2011). *Polyhedron*, **30**, 340–346.

[bb7] Murugavel, R. & Banerjee, S. (2003). *Inorg. Chem. Commun.* **6**, 810–814.

[bb8] Perrin, C. L., Lau, J. S., Kim, Y.-J., Karri, P., Moore, C. & Rheingold, A. L. (2009). *J. Am. Chem. Soc.* **131**, 13548–13554. 10.1021/ja905806h 19691344

[bb9] Sheldrick, G. M. (2008). *Acta Cryst.* A**64**, 112–122. 10.1107/S0108767307043930 18156677

[bb10] Yang, Y.-Y., Huang, Z.-Q., Szeto, L. & Wong, W. T. (2004). *Appl. Organomet. Chem.* **18**, 97–98.

